# Change of Expression of Ki 67, P53, and Cyclin D1 Immunoreactivity after *Helicobacter pylori* Eradication in Patients with Chronic Gastritis and Intestinal Metaplasia

**DOI:** 10.34172/mejdd.2025.444

**Published:** 2025-10-31

**Authors:** Seyed Mohammad Valizadeh Toosi, Somayeh Sheidaei

**Affiliations:** ^1^Gut and Liver Research Center, Non-communicable Diseases Institute, Mazandaran University of Medical Sciences, Sari, Iran; ^2^Department of Pathology, Faculty of Medicine, Mazandaran University of Medical Sciences, Sari, Iran

**Keywords:** Chronic gastritis, Intestinal metaplasia, *H. pylori infection*, Ki67, p53, Cyclin D1

## Abstract

**Background:**

We investigated the impact of *Helicobacter pylori* eradication on the expression of Ki67, p53, and cyclin D1 in patients diagnosed with chronic gastritis and intestinal metaplasia, utilizing the immunohistochemistry (IHC) method.

**Methods:**

The immunoexpression of Ki67, p53, and cyclin D1 in the gastric mucosa was analyzed in 26 patients with chronic gastritis, intestinal metaplasia, and confirmed *H. pylori* infection, as well as 10 patients with normal gastric histology and no *H. pylori* infection. The assessments were performed both before and after *H. pylori* eradication.

**Results:**

Successful eradication of *H. pylori* resulted in a significant reduction in the immunoexpression of Ki67, p53, and cyclin D1 in the majority of patients compared to pre-treatment. High immunoreactivity for Ki67, p53, and cyclin D1 was observed before eradication in 13 (50%), 4 (15.4%), and 9 (34.6%) patients, respectively. Following *H. pylori* eradication, none of the patients exhibited high immunoreactivity for these markers. Additionally, negative immunoreactivity for Ki67, p53, and cyclin D1 was noted in 21 (80.7%), 21 (80.7%), and 12 (46.1%) patients, respectively, with statistically significant P values of 0.005, 0.02, and 0.004.

**Conclusion:**

The eradication of *H. pylori* in patients with chronic gastritis and intestinal metaplasia leads to a significant regression in the immunoreactivity of Ki67, p53, and cyclin D1. This suggests the potential for reversing precancerous changes in the gastric mucosa through timely treatment.

## Introduction

*Helicobacter pylori* is one of the most widespread infections globally and plays a significant role in upper gastrointestinal disorders. It is a known cause of gastritis, gastric and duodenal ulcers, and, in rare instances, gastric lymphoma or cancer.^1^ Recent reviews on the prevalence of *H. pylori* infection indicate a declining trend among younger populations and in regions with higher socioeconomic development.^2^ Maleki and colleagues reported a prevalence of 44.5% of H. pylori infection among individuals in the general population.^3^ In another study conducted in our country, 66.6% of patients with dyspepsia were found to be infected with *H. pylori*.^4^ Eradication of *H. pylori* is considered a primary measure in preventing gastric cancer, particularly in regions with a high prevalence of the infection.^5-7^ According to Correa’s theory, chronic active gastritis caused by *H. pylori* infection can trigger a series of progressive changes in the gastric mucosa, potentially leading to gastric adenocarcinoma of the intestinal type.^8^ Intestinal metaplasia, characterized by the replacement of normal gastric mucosa with epithelium containing goblet and absorptive cells, is more commonly observed in its incomplete form.^9^

 Immunohistochemical (IHC) staining is a diagnostic technique that detects specific antigens by employing antibodies targeting cytoplasmic or nuclear components^10-12^ Ki67 is a nuclear protein present exclusively in proliferating cells, whereas p53 is a tumor suppressor gene responsible for regulating cell cycle arrest and apoptosis in response to DNA damage^13,14^ Cyclin D1, alongside associated proteins, governs cell growth, differentiation, survival, and death. Alterations in the expression of these cell cycle regulators are critical in the pathogenesis of cancers, including gastric cancer.^15,16^ This study aimed to assess the IHC expression of Ki67, p53, and cyclin D1 in patients with chronic gastritis and intestinal metaplasia before and after the eradication of *H. pylori*, with patients exhibiting normal gastric mucosa and no *H. pylori* infection serving as a negative control group.

## Materials and Methods

 This descriptive study included patients presenting with dyspepsia and alarming symptoms, or those aged over 45 years, who underwent upper gastrointestinal (GI) endoscopy. Patients with severe comorbidities, recent antibiotic or proton pump inhibitor (PPI) use, or a prior history of *H. pylori* eradication were excluded. All participants gave informed consent before participating in the study. During endoscopy, gastric mapping was conducted for all participants, which involved collecting two biopsies from the antrum, two from the body, and one from the incisura angularis. Pathology samples were examined by an expert pathologist, and patients diagnosed with chronic gastritis, intestinal-type metaplasia (complete and/or incomplete type), and *H. pylori* infection were included in the study.

 Among a total of 295 patients who underwent upper GI endoscopy, 59 patients had chronic gastritis and intestinal metaplasia. Of these, 19 patients tested negative for *H. pylori* infection and were excluded, resulting in 40 eligible participants. *H. pylori* eradication was performed using a 14-day quadruple therapy regimen, which included pantoprazole (40 mg), amoxicillin (1 g), clarithromycin (500 mg), and metronidazole (500 mg), administered every 12 hours.

 A follow-up endoscopy was performed at least 6 months after *H. pylori* eradication therapy. Biopsy samples were again collected using the gastric mapping protocol. Patients who continued to exhibit chronic gastritis and intestinal metaplasia in their pathology and were negative for *H. pylori* infection using the Giemsa staining method were included in the final analysis.

**Figure 1:**
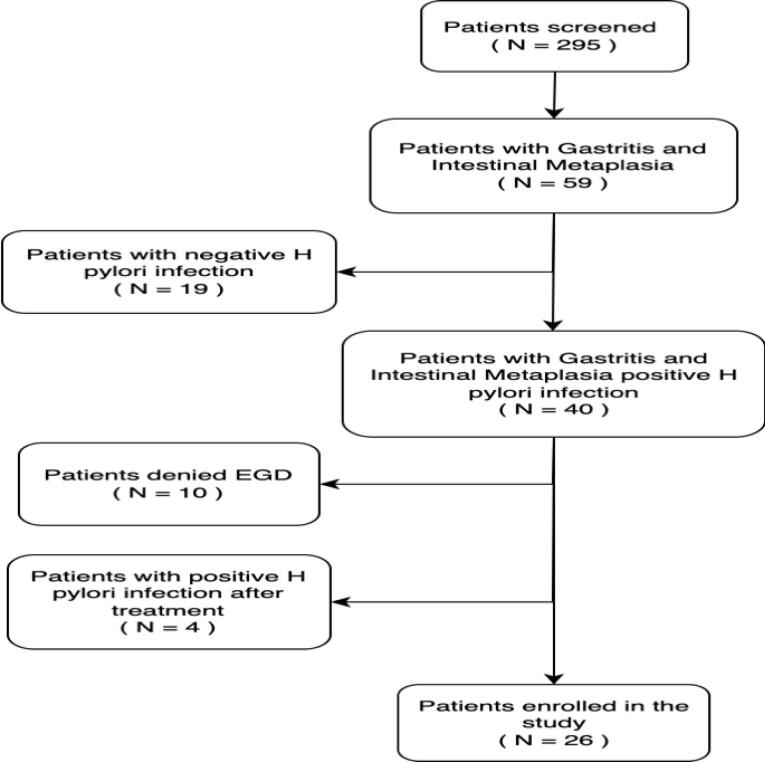
Flow diagram of patient selection and enrollment in the study

 Of the 40 initially enrolled patients, 10 withdrew due to unwillingness to undergo the second endoscopy, and four were found to still be positive for *H. pylori* after the second procedure. Ultimately, 26 patients with samples collected before and after *H. pylori* eradication were included for IHC analysis to evaluate Ki67, p53, and cyclin D1 expression (See Figure 1). Additionally, a group of 10 patients with normal gastric pathology or mild gastritis and negative for *H. pylori* infection was included as a control group for comparison.

###  Histology and Immunohistochemistry

**Figure 2:**
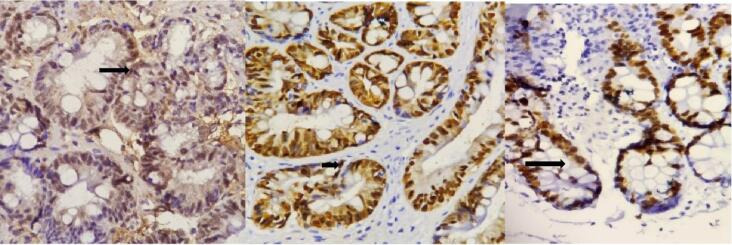
High IHC staining of cyclin D1 (nuclear), p53 (cytoplasmic and nuclear), and Ki-67 (nuclear) in a patient before *H. pylori* infection eradication. Arrows indicate positively staining cells. 400x magnification

**Figure 3:**
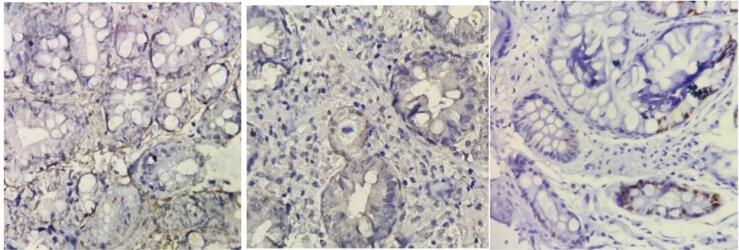
Negative and Low IHC staining of cyclin D1 (nuclear), p53 (cytoplasmic and nuclear), and Ki-67 (nuclear) in a patient after *H. pylori* infection eradication. 400x magnification

 To assess the impact of *H. pylori* eradication on the expression of Ki67, p53, and cyclin D1, formalin-fixed, paraffin-embedded tissue samples obtained from previously archived blocks in the pathology laboratory were used for IHC staining. The preparation process included deparaffinization, rehydration, and inhibition of endogenous peroxidase activity, followed by antigen retrieval, protein blocking, and the application of primary antibodies. The antibodies utilized in the staining process were Anti-Ki67 (clone GM010, mouse monoclonal, USA), Anti-p53 (clone BP-53-12, mouse monoclonal, USA), and anti-cyclin D1 (clone 28,970,002, rabbit polyclonal, USA). The presence or absence of brown color in the cell nuclei of the pathologic samples indicates the positivity or negativity of the histopathological markers of Ki67, p53, and cyclin D1, respectively (Figures 2 and 3).

 The percentage of nuclei with positive staining was determined by counting at least 500 nuclei per sample. Each IHC sample was evaluated using a semiquantitative scoring system, which assessed staining intensity (0: no stain, 1: weak, 2: medium, 3: strong) and the distribution of staining (0: < 5%, 1: 5–9%, 2: 10–19%, 3: 20–49%, 4: > 50%). The overall score was calculated by multiplying the percentage of stained cells by the intensity score, yielding a total score between 0 and 12. Scores were categorized as follows: less than 2 was considered negative (-), 3–5 indicated low expression ( + ), 6–8 moderate expression ( + + ), and greater than 9 high expression ( + + + ). This scoring system was derived from the study done by Dong and colleagues.^17^

## Results

 The study included 26 patients in the case group and 10 patients in the control group. Other demographic, endoscopic, and pathologic findings of patients are mentioned in Table 1. Among the participants, 15 (57.7%) were men and 11 (42.3%) were women. Prior to *H. pylori* eradication, high immunoreactivity was observed for Ki67, p53, and cyclin D1 in 13 (50%), 4 (15.4%), and 9 (34.6%) patients, respectively (Table 2). In contrast, low immunoreactivity for Ki67, p53, and cyclin D1 was noted in 13 (50%), 14 (53.8%), and 14 (53.8%) patients, respectively (Table 2).

**Table 1:** Demographic, endoscopic, and pathologic findings of patients in both study and control groups

	**Female (study group)**	**Male (study group)**	**Control group**
Number of patients	11	15	10
Average age	47/72	56/13	25/45
Positive history of smoking	0	3	0
Alcohol consumption	0	2	0
Endoscopic findings			
GU + pan-gastritis	1	2	0
Pan-gastritis	5	6	0
Antral-gastritis	6	5	2
Body-gastritis	0	1	1
Histopathologic finding			
IM in the antrum and body	6	5	0
IM in the antrum	5	8	0
IM in the body	0	2	0
*H. pylori* infection	11	15	0

**Table 2:** IHC staining immunoreactivity for cyclin D1, p53, and Ki67 of patients’ tissue samples before and after *H. pylori* eradication

**Tumor marker**	**Before HP eradication** **No. (%)**	**After HP eradication** **No. (%)**	***P*** ** value **	**Control group ** **No. (%)**
Ki67			0.005	
High	13 (50)	0 (0)		0 (0)
Low	13 (50)	5 (19.2)		2 (20)
Negative	0 (0)	21 (80.7)		8 (80)
p53			0.02	
High	4 (15.4)	0 (0)		0 (0)
Low	14 (53.8)	5 (19.2)		0 (0)
Negative	8 (30.8)	21 (80.7)		10 (100)
Cyclin D1			0.004	
High	9 (34.6)	0 (0)		0 (0)
Low	14 (53.8)	14 (53.8)		0 (0)
Negative	3 (11.5)	12 (46.2)		10 (100)

 Following the eradication of *H. pylori*, no patients exhibited high immunoreactivity for Ki67, p53, or cyclin D1. Low immunoreactivity for these markers was detected in 5 (19.2%), 5 (19.2%), and 14 (53.8%) of the tissue samples, respectively (Table 2). Additionally, negative immunoreactivity for Ki67, p53, and cyclin D1 was observed in 21 (80.7%), 21 (80.7%), and 12 (46.1%) of the patients’ samples, respectively.

 Immunoreactivity for Ki67, p53, and cyclin D1 was negative for all patients in the control group; however, weak positive immunoreactivity to Ki67 was found in two patients (Table 2).

 The statistical analysis, using McNemar and *t* test, showed significant reductions in the immunoreactivity of Ki67, p53, and cyclin D1 after *H. pylori* eradication, with *P* values of 0.005, 0.02, and 0.004, respectively. These findings highlight the impact of *H. pylori* treatment on reducing the expression of these markers.

## Discussion

 Our study revealed a significant reduction in the immunoreactivity of Ki67, p53, and cyclin D1 following successful eradication of *H. pylori* in patients with chronic gastritis and intestinal metaplasia. These findings provide strong evidence of the role *H. pylori* infection plays in promoting cellular proliferation and its potential to contribute to the neoplastic transformation of gastric epithelial cells. Key points from our findings are discussed below:

 The substantial decrease in Ki67, a proliferation marker, after *H. pylori* eradication indicates that the infection may drive increased cell turnover in the gastric mucosa, thereby elevating the risk of malignant transformation. The reduction of Ki67 expression to levels similar to those in the control group suggests that the gastric mucosa can return to a less proliferative, more stable condition following eradication. This underscores the importance of early detection and timely eradication of *H. pylori* in individuals at risk for gastric cancer. P53 is a tumor suppressor gene with multiple functions in cell cycle regulation and apoptosis.^18^ Ozturk and others evaluated dyspeptic children and found that 20.4% were in the p53-positive group, with a high proportion (91%) of these cases being associated with *H. pylori* infection, although intestinal metaplasia was not linked to p53 status.^19^ Another study on 31 *H. pylori*-positive patients who received eradication therapy showed a significant reduction in p53 immunostaining following successful treatment.^20^ In patients with chronic gastritis positive for *H. pylori*, p53 overexpression was observed in 15%, and after therapy, no patient in the *H. pylori*-eradicated group remained positive for p53. However, conflicting evidence exists; Berloco and colleagues found that among 75 patients with dyspepsia, only one exhibited p53 overexpression, which was unrelated to *H. pylori* status.^21^ Altered expression of cell cycle regulators is associated with tumor development and progression.^22^ Cyclin D1, which peaks during the G1 phase to facilitate DNA replication, is often overexpressed in gastric cancer and associated with poor outcomes.^23,24^ The cag pathogenicity island of *H. pylori* has been identified as a driver of cyclin D1 activation.^25^ One study in Iran by Mahmoudzadeh Sagheb et al evaluated the IHC expression of p53 and Ki67 in different gastric cancer, intestinal metaplasia, and dysplasia samples. They found that p53 expression was higher in *H. pylori*-positive samples across all three types, while Ki67 expression was higher only in intestinal metaplasia specimens.^26^

 The pronounced reductions in p53 and cyclin D1 immunoreactivity observed after *H. pylori* eradication are noteworthy. P53 mutations are frequently implicated in the progression of gastric cancer, while cyclin D1 dysregulation is commonly seen in cancer-related cell cycle abnormalities. The return of these markers to near-normal levels post-eradication highlights the potential to reverse precancerous conditions and prevent the development of gastric cancer.

 One study conducted in 2016 by Konstantinos Triantafyllou and colleagues evaluated the effect of *H. pylori* eradication on the expression of Ki67, p53, and cyclin D1, as well as on cell proliferation in gastric mucosa. Their findings mirrored ours, showing that successful *H. pylori* eradication restores the expression of these markers to levels comparable to controls. The potential clinical implications of our findings suggest that treatment of *H. pylori* not only eliminates the infection but also significantly reduces the expression of these markers, which may decrease the risk of gastric cancer in high-risk patients. This supports the recommendation for screening and treating *H. pylori* infection, particularly in patients with gastric mucosal changes such as chronic gastritis or intestinal metaplasia.

## Limitations of Study

 The limitations of our study include a relatively small sample size and the lack of long-term follow-up to evaluate the durability of marker reduction over time. Future research should focus on larger patient cohorts, longer follow-up durations, and the investigation of additional biomarkers to provide more comprehensive validation of these findings.

## Conclusion

 Our study offers significant insights into the role of *H. pylori* eradication in reducing or eliminating the expression of Ki67, p53, and cyclin D1 in patients with chronic gastritis and intestinal metaplasia. These results represent a promising step toward gastric cancer prevention and emphasize the importance of conducting larger-scale studies to validate these findings further and explore their clinical implications.
